# Personal

**Published:** 1909-11

**Authors:** 


### PERSONAL.

---

Dr. John A. Rafter, who has been living in the Orient for the past three years, has returned to Buffalo and resumed his relations with this JOURNAL, which were suddenly disrupted by his departure for the Philippines.

---

DR. JAMES FRANCIS RICE, of Buffalo, announces the removal of his office from The Kenilworth to 551 Elmwood avenue, one block north of Utica street. Telephones, Bell, Bryant 670, Frontier 758. Hours: Until 9, 2 to 3, 7 to 8. Sundays by appointment.

---

DR. WALTER B. CHASE, and Dr. Carroll Chase, of Brooklyn, have removed from 936 Saint Marks avenue to 1055 Park Place.

---

DR. MAUD J. FRYE, of Buffalo, an associate editor of this JOURNAL, who suffered ptomaine poisoning in the summer from which she was ill nearly three months, has recovered and resumed her professional practice at number 224 Allen street.

---

DR. WILLIAM H. MARCY, of Buffalo, announces that he has removed his offices and residence from 1148 Main street to 32 West Utica street.

---

DR. JOHN TINKLER, formerly of Cambridge Springs, Pa., has opened an office at No. 1847 Genesee street, for the general practice of medicine. He is a graduate of the Medical Department of the University of Buffalo, class of 1907.

---

DR. WILLIAM T. SHANAHAN, of Sonyea, who has been acting medical superintendent of Craig Colony for Epileptics for several months, has been made permanent head of the institution.

---

DR. CLARENCE L. HYDE, and DR. O. R. EICHEL, of Buffalo, have been appointed medical inspectors of tuberculosis, by the Commissioner of Health, Dr. Ernest Wende. The salary in each case is $1,000.

---

DR. W. G. GROVE, of 91 Niagara street, Buffalo, announces the removal of his residence to 723 Prospect avenue, and his offices to 87 West Genesee street. Hours, 8 to 10, 1 to 3, and 7 to 8.

